# Validation of Psychometric Properties of Partners in Health Scale for Heart Failure

**DOI:** 10.3390/jcm13237374

**Published:** 2024-12-03

**Authors:** Pupalan Iyngkaran, David Smith, Craig McLachlan, Malcolm Battersby, Maximilian De Courten, Fahad Hanna

**Affiliations:** 1 Melbourne Clinical School, University of Notre Dame, Melbourne, VIC 3029, Australia; pupalan.iyngkaran@student.torrens.edu.au; 2 Centre for Healthy Futures, Torrens University Australia, Surry Hills, NSW 2010, Australia; craig.mclachlan@torrens.edu.au; 3 Program of Public Health, Department of Health and Education, Torrens University Australia, Melbourne, VIC 3000, Australia; 4 Collège of Medicine and Public Health, Flinders University, Adelaide, SA 5000, Australia; david.smith@flinders.edu.au; 5 SALHN Mental Health Service, Flinders Health and Medical Research Institute, College of Medicine and Public Health, Flinders University, Adelaide, SA 5000, Australia; malcolm.battersby@flinders.edu.au; 6 Mitchell Institute, Victoria University, Melbourne, VIC 3000, Australia; maximilian.decourten@vu.edu.au

**Keywords:** congestive heart failure, Flinders program, PIH scale, risk assessment, self-management

## Abstract

**Background:** Congestive heart failure (CHF) is a complex chronic disease, and it is associated with a second comorbid condition in more than half of cases. Self-management programs can be specific to CHF or generic for chronic diseases. Several tools have been validated for CHF. Presently, there are no established generic instruments that are validated for measuring self-management in CHF. **Objective:** This study aims to evaluate the internal reliability and construct validity (psychometric properties) of the Partners in Health (PIH) scale for patients with congestive heart failure, a generic chronic disease self-management tool. **Methods:** The study included 210 adult CHF patients [120 with heart failure with reduced ejection fraction (HfrEF), 90 with preserved ejection fraction (HfpEF)], from Community Cardiology Outpatients in West Melbourne, Australia, who were treated in community cardiology and were included between May 2022 and Jan 2024. The screened patient population were diagnosed with CHF and were eligible for an SGLT-2 inhibitor. Cohort analysis used the Bayesian confirmatory factor analysis to evaluate the a priori four-factor structure. Omega coefficients and 95% credible intervals (CI) were used to assess internal reliability. **Results:** In the CHF (HFrEF) and preserved ejection fraction (HFpEF) cohorts, participants’ mean [standard deviation (SD)] age was 66.8 (13.5) and 71.3 (9.76) years. Description of study sociodemographics highlighted that 88% and 52% of patients were male, there was a BMI > 50% in both cohorts, eGFR > 60 mL/min were 59% and 74%, and LVEF < 40% and > 50% were 99% and 100%, respectively. Model fit for the hypothesised model was adequate (posterior predictive *p* = 0.073) and all hypothesised factor loadings were substantial (>0.6) and significant (*p* < 0.001). Omega coefficients (95% CI) for the PIH subscales of Knowledge, Partnership, Management and Coping were 0.84 (0.79–0.88), 0.79 (0.73–0.84), 0.89 (0.85–0.91) and 0.84 (0.79–0.88), respectively. **Conclusion:** This study is original in confirming the dimensionality, known-group validity, and reliability of the PIH scale for measuring generic self-management in outpatients with CHF syndrome.

## 1. Introduction

Congestive heart failure (CHF) is a chronic disease. Chronic disease self-management (CDSM) is important for CHF patients for the following two reasons. Firstly, in Australia, at least 50% of the population suffers from one of these eight chronic conditions, namely, heart disease, diabetes mellitus, arthritis and back pains, mental health conditions, asthma, chronic obstructive pulmonary diseases and cancer [1]. CHF in many cases is associated with at least one of the chronic conditions above [2]. Secondly, the prevalence, morbidity, mortality, readmission and resource cost of CHF are on the rise. CHF is resource intensive, and its disease loads and hospital readmissions continue to rise [3,4]. While empowering patients through chronic disease self-management has a sound theoretical basis, the CHF guidelines are complex, and there are unanswered questions and evidence gaps [5].

An important theoretical framework, Wagners ‘Chronic Care Model’ [6], published several decades ago, identified two key domains and six key elements: the health system domain which requires organised health services, delivery system redesign, clinical information systems and decision support; and a community domain requiring community linkages to resources and policies to enhance self-management support. Cameron et al. [7], in 2009, reviewed 14 established CHF-specific instruments, that were promoted into best practice, for the provision of disease-specific measures of CHF self-care behaviours; psychometric analysis revealed that only 2 tools were reliable and valid to specifically measure CHF self-management. In the ensuing decades, publications have covered a range of topics from theory, practical management, and consensus guidelines, with optimism [8,9,10,11,12]. Nonetheless, trial results were not forthcoming, and the American Heart Foundation demoted self-management in CHF from a performance to a quality measure [10], which has filtered into the guidelines [3,4,5]. Thus, the purpose of this paper is to create a focus on generic CDSM programs and build the evidence from this platform. The program in review is the Flinders Program of Chronic condition Self-management (CFPI), which is a gold standard tool for CDSM. The CFPI has been validated, published data support its good validity and internal consistency, and it is also utilised clinically across a range of conditions [1,13,14,15,16,17,18,19,20,21,22,23]; however, it is not for CHF. The basis of its theoretical foundation was published in 2003, where the Partners in Health (PIH) scale, a patient-reported outcome tool using 12 questions and over 4 domains, assessed patients’ skills (knowledge, partnership, recognising and managing, coping) related to symptoms and their condition. This paper uses Battersby’s et al. definition of self-management as ‘*the active involvement of the patient in the management of their chronic medical condition*’ [14].

In this study, we aim to assess the psychometric properties (internal reliability and construct validity) of the Partners in Health (PIH) scale, a chronic condition self-management instrument to assess baseline self-management behaviours in a CHF cohort. For this syndrome, the literature supports the observation that validated CHF-specific CDSM programs are few and comorbid conditions are frequent in CHF [5,24]; thus, assessing both disease-specific and generic CDSM tools could be important, and for the generic Flinders Program of CDSM, validating the PIH scale is an important first step in utilizing this tool.

## 2. Methods

The study data are from a subanalysis of the *SELFMAN*agement in Heart Failure Study (*SELFMAN-HF*) study, as described in the protocol [24].

### 2.1. Design

A clinical audit was performed on 210 consecutive patients screened for the *SELFMAN-HF* study, assessing the efficacy of CDSM programs, in patients commencing Sodium Glucose Co-transport 2 inhibitor (SGLT-2i) for CHF. As a routine for chronic disease management, all patients completed the PIH tool at baseline. The cohort included patients with heart failure with reduced ejection fraction (HFrEF) and heart failure with preserved ejection fraction (HFpEF). The study team and expert panel designed the study, and oversaw the conduct and analysis of results. The analyses were conducted by 3 investigators (PI, FH, DS).

### 2.2. Participants

For this audit, eligible patients were those aged over 18 years who were screened for the use of an SGLT-2i within May 2022 and Jan 2024, with a clinical diagnosis of CHF, who were eligible for an SGLT-2i and had completed a PIH assessment as a routine clinical work-up and care. Patients screened were predominantly a HFrEF cohort, who were aged over 18 years and started SGLT-2i therapy within 6 months for systolic CHF indication, with an echocardiographic left ventricular ejection fraction (LVEF) of <40%. Patients screened with a LVEF > 40%, who completed the PIH scale at baseline were not enrolled into the prospective *SELFMAN-HF* study; however, baseline data were used to form this psychometric audit analysis. Patients were excluded if concerns were raised by any medical staff, if they had a life expectancy of ≤6 months whilst receiving palliative or nursing home care, or if they had a significant cognitive impairment, or if they had started an SGLT2-i >6 months prior [24].

### 2.3. Sample Size Calculations

The effect size for the introduction of SGLT-2i in a community cardiology population with treated CHF is not known. The study is designed to enrol a minimum of 80 patients with LVEF <40% and a New York Heart Association (NYHA) class of at least 2 or 3. The documented event rates in similar settings for readmission and mortality at 1 month and 1 year were 25%. Thus, a follow up at 12 months would see >100 admissions and >10 mortalities. After interim analysis at 3–6 months, and due to the lower event rates, the sample size was increased to the documented number.

### 2.4. Trial Instruments and Procedures

#### 2.4.1. CFPI Program and PIH Tools

The CFPI is designed as a generic, comprehensive CDSM program that contains various tools (questionnaires) to harness a detailed patient-centred, comprehensive and flexible program for chronic disease assessment and management. This tool is the gold standard and is on par with other programs in achieving its outcomes, i.e., assessing self-management understanding and goals, and from this understanding, we can tailor education to achieve self-efficacy for managing chronic disease. The CFPI program scores patient-reported outcomes (PRO) and extrapolates health staff input with health services data along 4 parts [13]: (1) The Partners in Health (PIH) scale is a self-rated questionnaire for the patient to assess their self-management knowledge, attitudes, behaviours and the impacts of their chronic condition. (2) The PIH scale uses 12 questions and an 8-point Likert scale to score a patient on 4 self-management domains, knowledge, partnership in treatment, recognition and management of symptoms and coping; (3) the additional parts, including the cue and response, problems and goals and chronic condition care plan, of the (4) CFPI are detailed in these references [1,13,14,15,24,25,26,27,28,29,30,31].

#### 2.4.2. Data Collection

The PIH scale was completed by all patients screened at the heart failure clinic. Forms with completed in the waiting room, the doctors’, or the heart failure nurse’s office. Patients were offered guidance if they had difficulty interpreting the study and asked for assistance. The PIH scale uses Likert-type scales (an 8-point rating scale), which allow change and progress to be measured and recorded during reviews. The PIH scale is provided to the patient in the waiting room or the consulting room. Time is provided to fill in the scale. Patients are provided assistance if they are not able to understand or if they have a carer or partner to help them.

#### 2.4.3. Ethical Considerations

The SELFMAN-HF study was conducted accordingly according to the guidelines set out by Declaration of Helsinki. The study received approval from the Institutional Review Board (or Ethics Committee) of the St Vincent’s ethics committee (approval no. LRR 177/21) with a date of approval of 17 May 2022. All patients who participated, prior to their enrolment, read and completed written informed consent forms.

#### 2.4.4. Statistical Aspects and Data Analysis

We investigated the convergent and discriminant validity of the PIH scale using Bayesian Confirmatory Factor Analysis (BCFA). This approach is a compromise between maximum likelihood (ML) CFA and exploratory factor analysis (EFA). In a single step analysis, BCFA enables the specification of the prior hypothesized major factor patterns as well as informative small-variance priors for cross-loadings and residual covariances. The cross-loadings model the relationships between PIH indicators and nontarget factors, and residual covariances model shared sources of influence on the PIH indicators that are unrelated to the factors. A strategic use of such priors facilitates a more substantive interpretation of model parameters in the context of the current study population. This approach also minimizes the capitalization on chance that otherwise may occur through a sequence of model modifications. Statistical inferences about parameter estimates and model fit are made from a posterior distribution. A BCFA is like a standard CFA but does not rely on large-sample theory and performs better with small samples compared to ML algorithms. In other words, the Bayesian framework does not require traditional model identification to estimate model parameters. Previous studies have shown that using prior information (e.g., small variance priors for cross-loadings and residual covariances) in a Bayesian estimation framework provide reliable estimates for small study samples (e.g., n= 20; n = 40).

Bayesian confirmatory factor analysis was first used to estimate the PIH-CFA model with zero cross-loadings. We then repeated the BCFA, firstly with the addition of small, informative priors for cross-loadings and then with additional non-zero priors for the residual correlations. The observed variables were standardized in accordance with the standardized priors. To identify a suitable cross-loading prior variance, a range of increasing values (0.001, 0.005, 0.01, 0.15, 0.20, and 0.30) was initially tested. The aim was to find a value that made a substantial reduction in the 95% confidence interval for the difference between the observed and the replicated chi-square values relative to the original CFA model without cross-loadings whilst not sacrificing the speed of convergence [32,33,34,35,36,37,38,39,40,41,42].

The Inverse-Wishart (*IW*) distribution was then used for the addition of residual correlations to the model. This is the conventional prior distribution for covariance matrices in Bayesian analysis [32]. Priors were set for *IW*(*dD*, *d*), where *d* is the degrees of freedom of the distribution and *D* is a diagonal matrix comprising residual variances from the BCFA model [32,33]. The starting value for the degrees of freedom parameter was set at *d* = 50 and then varied to find the largest value that generated a posterior predictive *p*-value (PP *p*) greater than 0.05 to show an acceptable BCFA model fit that was, at the same time, close to the CFA model. This was carried out to enable the identification of the smallest possible modifications to address CFA model misfit [32]. BSEM does not require the assumptions of normality and is independent from large sample theory [33].

Bayesian estimation comprised 8 independent Markov chain Monte Carlo (MCMC) algorithms and two processors. The minimum number of iterations was set at 15,000 with a maximum of 100,000 and was monitored using the potential scale reduction (PSR) criterion where values less than 1.1 provide evidence for convergence [34]. To evaluate BCFA models, Bayesian versions of approximate fit indices and root mean square errors of approximation (RMSEA), the confirmatory fit index (CFI) and Tucker–Lewis index (TLI) were used. The suggested cut-off values for reasonably well-fitting models are RMSEA < 0:06, TLI > 0:95 and CFI > 0:95. The Discrepancy Information Criterion (DIC) was also used to compare models as it takes into account model complexity via the estimated number of parameters or effective number of parameters [32,35]. Lower values are indicative of a better fitting model. To assess the internal reliability of PIH raw scores, McDonald’s omega coefficients and 95% Bayesian credible intervals were calculated using estimates from the final model [36]. For health status questionnaires, coefficient values between 0.70 and 0.95 are indicative of good internal consistency [37]. All analyses were conducted using MPlus software (Version 8.9).

## 3. Results

### 3.1. Patient Demographics and Characteristics

**Figure 1:**
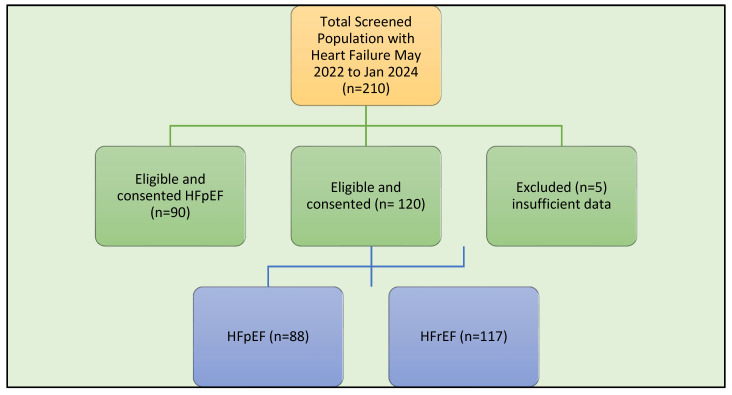
Flowchart. HFpEF—heart failure with preserved ejection fraction; HFrEF—heart failure with reduced ejection fraction; n = number.

From May 2022 to January 2024, 210 CHF patients (117 with HFrEF and 88 with HFpEF) were audited from a cohort that was eligible for SGLT-2i who had completed the PIH scale and had (Figure 1) available baseline information.

### 3.2. Baseline Characteristics (HFrEF)

The HFrEF population cohort comprised 117 patients, and the baseline study characteristics are summarized in Table 1. The mean age was 66.8 years old (SD 13.5), regarding gender, 88 (75%) identified as male and 29 identified as female. The ethnicity of the majority of study patients was Caucasian (90 patients (77%)), followed by South Asian and Asian, African and Indigenous Australian. Most patients were married, totalling 75 patients (64%). For family support, at least 71 patients (61%) described spouse or family supports for HF and chronic disease. For background studies, 63 (54%) patients had education up to a high school level. Renal impairment is defined as an eGFR of <60 mL/min, and this was recorded in 48 (39%) patients. At baseline, 39 patients (25,6%) in the study cohort did not reveal or have records of any associated comorbidity, while 26 (22.3%) had one and the other participants had three or more. Specifically, hypertension and hypercholesterolemia were the most common comorbidity in 79 (68%) and 73 (62.4%) patients, respectively. Other common comorbidities were coronary artery disease (CAD), diabetes and obstructive sleep apnoea, which were recorded in 51 (44%), 42 (36%) and 31 (26.5%) of the patients in the cohort, respectively. A history of tobacco use was recorded in 53 patients (45%). The diagnosis of CHF was not new, i.e., at baseline, it was a chronic condition, wherein 34 (29%) of the cohort diagnoses were made prior to 12 months. As per the inclusion criteria, at baseline all patients had been prescribed and had commenced SGLT2-i for no more than 6 months. At enrolment, 3 of 4 CHF [beta-blocker, renin–angiotensin–aldosterone and/or neprilysin inhibitor (RAAS/ARNI) or mineralocorticoid receptor antagonist (MRA)] pillars were already started in participants. Most of these participants who had received a diagnosis of CHF within or in a greater than 12 month period were also optimised on CHF pillar therapy, consistent with guideline requirement for eligibility for SGLT-2i, where all patients were an NYHA class 2 or more, with left ventricular ejection fraction (LVEF) Class III (<40%) and greater. CHF patients were also on an antiplatelet or anticoagulant at baseline for CAD, AF or other comorbidities. The CHF-specific pillar therapies were 112 (96%) for BB, 111 (95.1%) for RAAS/ARNI, and 46 (39%) for MRA. Polypharmacy was noticeable and common with CHF and comorbid conditions, where 101 patients (68%) had at least five prescribed medication classes. Cardiac rehabilitation was only recorded among 16 patients (14%).

### 3.3. Baseline Characteristics (HFpEF)

The study cohort with HFpEF comprised 88 patients, and their baseline characteristics are summarised in Table 1. The population was 71.3 years old (SD 9.76); 46 patients (52%) were male and 42 (48%) were female, with a high amount of very distinct demographics for HFrEF. Regarding the ethnicity, the majority of the cohort was Caucasian [90 (77%)], followed by South Asian, Asian, African or Indigenous Australian. Most patients were married [75 (85%)]. Patients reported having support, as at least 71 patients (82%) stated that their spouse or family supported their illness needs. A majority (63 patients (71%)) of patients had education up to a high school level. Renal impairment was defined as an eGFR of <60 mL/min, and was recorded in 23 patients (26%). A handful of patients (3 patients (3.5%)) did not report associated comorbidities, while 32 patients (36.3%) had one and the others had three or more. Hypertension and hypercholesterolemia were the record as the most prevalent comorbidities, with 76 (87%) and 67 (76%) patients, respectively, reporting these. Coronary artery disease (CAD) and diabetes were recorded in 30 (41%) and 29 (33%) patients, obstructive sleep apnoea was also common, recorded in 21 patients (24%) in the cohort. A smoking history was recorded in 29 patients (33%). A prior CHF diagnosis (i.e., a chronic condition, having been diagnosed prior to 12 months) was reported in only 7 patients (8%) from the cohort. At baseline, 71 (80.7%) patients had commenced taking Sodium Glucose Co-Transporter-2 Inhibitors (SGLT2-i), within 6 months of identification for study enrolment. At this point, with HFpEF, the heart failure pillar was not relevant and only 2 patients (2.3%) were on ARNI, 46 (52.3%0 on BB, and 15 patients (17%) were on MRA, which was starkly different to the HFrEF cohort. Patients with a diagnosis of CHF within the last 12 months had commenced CHF pillar therapy (BB, RAAS/ ARNI or MRA). Regarding the eligibility for SGLT-2i in HFrEF, all patients were an NYHA class 2 or more; however, all patients had LVEF Class 1 (>50%), which was a clear separation for the HFrEF patients. CHF therapy was not as good where diuretic, MRA and calcium channel blocker (CCB) use were lower. The use of an antiplatelet or anticoagulant at baseline varied based on comorbidity. CCF pillars for HFpEF include only the SGLT2i and were used in 71 patients (80.7), reflecting difficulty in the diagnosis and ongoing understanding of HFpEF treatments and overlap with other indications, such as DM, and proteinuric chronic renal impairment. Polypharmacy was common, and 27 patients (31%) had at least five prescribed medication classes, although this was lower than that for HFrEF. Cardiac rehabilitation was not recorded in any participant for HF.

### 3.4. BCFA Model

The BCFA with exactly zero cross-loadings and residual correlations corresponding to the pre-specified regular CFA model provided a posterior predictive *p*-value of almost zero (Table 2). The specification of small (close to zero) informative variances for cross-loadings ranging from 0.001 to 0.03 did not lead to an acceptable model fit based on PP *p* < 0.001. The addition of residual correlations produced almost perfect model fit for a larger prior (d = 50), as shown by PP *p* values being close to 0.5 and confidence intervals being symmetric about zero. Table 2 results show acceptable model fit for small informative cross-loadings of 0.005 and residual correlations d = 200 as indicated by goodness of fit indices whilst being close to the BCFA-based CFA model (PP *p* = 0.144). Table 3 shows factor loadings and factor correlation estimates for the BCFA and CFA model. All cross-loadings for the BCFA model were less than or equal to 0.1 and statistically non-significant in that 95% Bayesian credibility intervals covered zero. All hypothesised factor loadings were substantial and significant at *p* < 0.001 and factor correlation estimates were in the moderate range (0.409–0.662). For estimates of residual correlations, 95% (63/66) were statistically non-significant in that 95% Bayesian credibility intervals covered zero. Three residual correlations were statistically significant though ignorable in terms of statistical magnitude (<0.14) and practical implications. In conclusion, posterior predictive assessments of model fitness showed mostly ignorable discrepancies between BCFA and CFA models from a statistical and practical perspective. This provided additional support for a PIH four-factor solution using a standard CFA approach.

To assess the reliability of subscale scores for the BCFA four-factor model with small variance priors, McDonald’s omega coefficients were calculated using unstandardised factor loadings and item residual variances. Coefficients (95% Bayesian credible intervals) for the PIH subscales of knowledge, partnership, management, and coping were 0.84 (0.79–0.88), 0.79 (0.73–0.84), 0.89 (0.85–0.91) and 0.84 (0.79–0.88), respectively. These values indicated that the subscales’ internal reliability in producing raw scores was in the acceptable range [32,33,34,35,36,40].

## 4. Discussion

In this study, a generic patient-reported self-management measure, the PIH scale, is validated for CHF. Prior to this, only two measures of self-management, both of which are disease specific [Self-care Heart Failure Index (SCHFI) and European Heart Failure Self-care Behaviour Scale (EHFScBS)] and have rigorous validity and reliability, were used for psychometric testing in CHF [7]. The PIH scale was developed at a time where evidence was building for the effectiveness of CDSM, yet there were no instruments to assess its effectiveness [13,14]. The findings we present here are encouraging and advance the investigation of the psychometric properties of the PIH scale, which is validated across many languages and chronic conditions [1,13,14,15,25,26,27,28,29,30,31], and is now, for the first time, purposed for the spectrum of CHF syndromes.

### 4.1. Summary of PIH and Theoretical Framework of Generic Self-Management Scale

In their seminal paper, Battersby et al. [14] showed that the PIH scale can be completed by patients as a patient-reported outcome (PRO or patient self-rating) tool and interpreted by health professionals as a marker of self-management capabilities to facilitate patients’ care. This preliminary investigation of the psychometric properties of the PIH scale supported its face validity among patients, general practitioners, and other health professionals (e.g., nurses), and its concept validity in foundations based on sound self-management definitions. The study also demonstrated reliability in both internal consistency and inter-rated reliability. The PIH structure then underwent factor analysis, and revealed three stable and meaningful factors underlying the overall scale: core self-management, condition knowledge, and symptom monitoring [14]. The authors themselves highlighted this sentinel work as preliminary due to the small sample size of 46 patients. Nonetheless, the authors raised important points in the summation for future research including increasing the sample size and using other components of the scale such as the health-worker-prompted Cue and Response to improve the accuracy of PRO.

Since then, studies of diabetics, arthritis, chronic lung diseases, chronic liver disease, and heart failure (including this study), and those of such conditions that have been conducted across multiple languages have supported these original claims. The theoretical framework is validated, rigorously tested and has held up across a range of chronic conditions’ self-management [1,13,14,15,25,26,27,28,29,30,31], and it also has relevance for deficiencies reported in CDSM and CHF [5,11,12]. The PIH scores patients across four self-management domains (knowledge, partnership in treatment, recognition and management of symptoms, and coping) to develop a baseline of self-monitoring, self-tailoring and self-efficacy abilities. How this translates for generic chronic conditions versus disease-specific conditions is an ongoing question.

### 4.2. Summary of Self-Management in Heart Failure and Comparing Relevant Research

A lot has changed in the CHF landscape. Today, comprehensive care (including rehabilitation) around guideline-derived medical therapies (GDMT) [3,4] is delivered through an organised process, which ensures the timely and high uptake of prognostic therapies for stable patients. This is further supported during decompensation. This care improves all major cardiovascular events (MACEs). The CHF syndrome has chronological characteristics, and the disease trajectory has changed with the rapid therapeutic developments over the last several decades.

Nonetheless, the argument for self-management holds for CHF. In the first decade of this millennia, some important observations were highlighted. Riegel et al. studied 2080 adults from developed (United States, Australia) and developing (Thailand and Mexico) nations using the Self-Care of HF Index (SCHFI); all measures of self-management were inadequate, although these were higher in the developed nations. This study concluded that self-management interventions were greatly needed across all groups [8]. Importantly at this stage, with 14 available clinical instruments to assess self-management, only two were reliable and valid for measuring CHF self-management [7]. Practical recommendations for the self-management of CHF followed, which covered a generic range of health topics within a CHF context. The most specific aspects included symptom recognition and fluid and sodium management [9]. With regard to the research and translation of CDSM, there are confounding characteristics that affect self-management capabilities including comorbid conditions, psychological (mood, anxiety, cognitive and sleep disturbance) and physical functional status, and demographics (health literacy, social isolation and support, socioeconomics). These factors could influence behavioural more than pharmacological therapies, the later of which also receives greater emphasis in education and delivery.

The evidence that was published in that era, despite the above issues that needed to be factored in, entailed encouraging trends, but without definitive gold standard results. These covered a range of outcomes reproducing optimal GDMT uptake [43,44,45,46,47,48,49], improving readmissions and MACEs [50,51], event-free survival [52,53,54,55,56], and age, quality of life and functional status [57,58,59,60,61]. Feng et al. performed a meta-analysis of 20 randomised controlled trials (7 rated Grade A and 13 Grade B), enrolling 3459 patients with CHF, and showed that administered CDSM programs improved patient skill, quality of life, and readmission rates, although this did not extend to mortality [60].

Regarding study size, only four studies enrolled more than 200 patients, with at least 100 in control or treatment portions. The follow-up periods of the included studies ranged from 6 to 12 months. The CDSM intervention delivered varied in location, health staff, duration and intensity [60]. Thus, the statements for authoritative consensus on this topic remain accurate. The early positions in 2009 and 2017 by Reigel et al. included that “…Although there are many nuances to the relationships between self-care and outcomes, there is strong evidence that self-care is effective in achieving the goals of the treatment plan and cannot be ignored. As such, greater emphasis should be placed on self-care in evidence-based guidelines…”, and these remain accurate [8,9,11]. However, the nuances remain; while great emphasis was encouraged, it is understandable that, unlike the pharmaceutical and device trials, without consistent baseline CDSM program standards in its delivery and follow-up, it is not feasible to achieve the Grade 1A or gold standard finding to comment on MACEs, of for current consensus statements to be provided [3,4].

Specifically related to the Flinders Program (*FP*), this study has shown that the generic CDSM principles are valid. It has been many decades since the establishment of Wagner’s Chronic Care Model and the *FP*; nonetheless, the domains described in the previous paragraph remain relevant as surrogates of patient perceptions of behaviour and are valid. The next steps in integrating generic and disease-specific tools are topics for future studies.

### 4.3. Current Findings and Future Research

The findings from this study are promising and provide support for advancing further research into self-management and self-management support in CHF. Firstly, it does appear that a generic tool can be utilised in a heterogenous population of patients with HFrEF, HFpEF, a high comorbidity burden and a diverse demography with good internal validity. This ticks an important box for self-administered assessments for CDSM instruments. Alongside this paper, we have completed a study in HFrEF which explores the external validity of this tool. The details are discussed further in another paper [24].

It is interesting to note the simplicity of the concepts, as they flowed firstly from base findings on knowledge and CDSM efficacy. These results are consistent with the theoretical proposition that limited education interventions do little to influence health-related behaviours and skills. The PIH scale is part of a more comprehensive Flinders CDSM program. The Cue and Response form is useful tool to follow the PIH scale in identifying any deficits/issues at the beginning and end of an assessment. The PIH scale is important in gauging a patient’s perspective, so long as they understood the scale and questions. This simple and complex link in these tools is an area of future importance. Also important is the role of dyads in CDSM. The dyads are the patient and health professionals and the patient and their dedicated carer in the journey of assessing their baseline and progress (judging scales and levels), with the intention of achieving an agreed knowledge, forming partnerships, planning, symptom monitoring and management and coping. This internal validation leaves room to expand on the clinical bedside workings of this generic CDSM tool.

### 4.4. Limitations

The study presents data from a single clinic from a consecutive screened and unran-domized population of patients with HFrEF and HEpEF. Patients who had HFpEF have some gaps in baseline social data. These comorbidities and medications are relevant in broad discussions of CDSM [62]. While not relevant for assessing preliminary internal validity, broader demographic, cultural and comorbidity samples will be required to confirm the study findings. greater trial controls will be required for future works. Questions of concurrent validity, reliability, and reproducibility in a larger multisite clinical population of CHF require more advanced study designs including randomisation and follow-up data to assess whether the PIH scale is a valid measure of change in self-management, clinical and quality-of-life outcomes over time.

## 5. Conclusions

In this study, we describe the first validation of the PIH scale, on the spectrum of heart failure syndromes (HRrEF and HFpEF). The PIH scale, a component of the Flinders Program, is validated across a range of chronic diseases and now for CHF. This study has confirmed the reliability, validity and dimensionality of the PIH scale as a patient-reported tool for measuring self-management capabilities for key domains, including collective self-efficacy for the CHF syndrome. For the next step, in other chronic diseases, studies have shown that the translation of patient scores, to tailored self-management support with the CFPI, improves patients’ knowledge, skills for their disease and their case management. This study lays a foundation to assess this concept for CHF. Importantly, it also raises questions of the steps required to introduce CHF (disease-specific) aspects within the generic program. The traditional CHF approach has been predominately disease specific. This is an area that requires further exploration in the future.

## Figures and Tables

**Table 1:** Baseline sociodemographic and clinical variables.

Variable	HFrEF n = 117	%	HFpEF n = 88	%
Age (yo)	66.8 mean	SD 13.5	71.3	SD 9.76
Sex, (men)	88	75	46	52
Ethnicity				
Caucasian	90	77	71	81
South Asian	7	6	6	7
Asian	3	3	4	4
African	10	8	6	7
Aboriginal/Pacific Is	7	6	1	1
Social			NA	NA
Married	75	64		
Spouse support	71	61		
High School	63	54		
Smoking history				
No	53	45	59	67
Ex/Yes	64	55	29	33
Comorbidities				
CRF (eGFR mL/m)				
>60	69	59.0	65	74
30–60	39	33.3	17	19.3
15–30	7	6.0	6	6.7
<15	2	1.7	0	0
CAD	51	44.0	30	41
DM	42	36.0	29	33
HT	79	68.0	76	87
Chol	73	62.4	67	76
OSA	31	26.5	21	24
Years with HF diagnosis				
Less than 1 year	83	71	81	92
1–4 years	21	18.0	7	8
5–10 years	13	11.0		
LVEF				
Grade 1 (>50%)	0	0	88	100
Grade 2 (40–49)	1	0.8	0	0
Grade 3 (30–39)	84	71.2	0	0
Grade 4 (20–29)	29	25	0	0
Grade 5 (<20)	4	3	0	0
NYHA classification at discharge				
I	0	0	0	0
II	73	62.4	68	77
III	41	35.0	20	23
IV	3	2.6	0	0

Abbreviations: CAD—coronary artery disease; CRF—chronic renal failure; DM—diabetes mellitus; eGFR—estimated Glomerular Filtration rate; Ex—ex-smoker; HT—hypertension; HFrEF—heart failure with reduced ejection fraction; HFpEF—heart failure with preserved ejection fraction; Is—island; LVEF—left ventricular ejection fraction; n—number of participants; NA—not available; NYHA—New York Heart Association; OSA—obstructive sleep apnoea; SD standard deviation; yo—years old.

**Table 2:** Model fit statistics for Bayesian confirmatory factor analyses.

			Difference Between Observed and Replicated χ^2^ 95% CI CI							
PIH Model	PPP *p*	PP *p*	Lower 2.5%	Upper 2.5%	# Par.	pD	DIC	RMSEA (90% CI)	CFI (90% CI)	TLI (90% CI)
CFA		<0.001	128.5	213.3	42	47.1	2987	0.189 (0.178–0.206)	0.836 (0.804–0.855)	0.748 (0.699–0.777)
*BCFA with cross-loadings*										
xload N (0, 0.001)	<0.001	<0.001	80	151.3	78	46.5	5605	0.117 (0.107–0.127)	0.913 (0.897–0.928)	0.869 (0.843–0.890)
xload N (0, 0.005)	0.007	<0.001	44.5	119.7	78	52.9	5577	0.107 (0.092–0.121)	0.938 (0.920–0.954)	0.890 (0.858–0.918)
xload N (0, 0.01)	0.121	<0.001	31.4	106	78	56	5568	0.103 (0.087–0.118)	0.948 (0.930–0.963)	0.898 (0.864–0.927)
xload N (0, 0.015)	0.304	<0.001	28	101.3	78	57	5565	0.101 (0.085–0.117)	0.951 (0.934–0.965)	0.901 (0.867–0.930)
xload N (0, 0.02)	0.451	<0.001	26.7	99.9	78	58	5563	0.100 (0.084–0.116)	0.952 (0.936–0.966)	0.903 (0.869–0.932)
xload N (0, 0.03)	0.654	<0.001	26.1	97.8	78	56	5560	0.097 (0.081–0.114)	0.953 (0.937–0.967)	0.908 (0.876–0.936)
*BCFA with cross-loadings and residual covariances*										
xload N (0, 0.005) res corr (d = 50)	0.858	0.493	−37.7	37.6	144	73	5515	0.023 (0.000–0.088)	0.999 (0.981–1.0)	0.995 (0.925–1.0)
xload N (0, 0.005) res corr (d = 100)	0.776	0.330	−29.3	46.4	144	67	5517	0.047 (0.000–0.087)	0.993 (0.974–1.0)	0.979 (0.927–1.0)
xload N (0, 0.005) res corr (d = 200)	0.545	0.144	−17.4	59.4	144	60	5522	0.061 (0.014–0.089)	0.984 (0.965–0.999)	0.965 (0.924–0.998)
xload N (0, 0.005) res corr (d = 300)	0.382	0.072	−10.1	67.3	144	56	5527	0.067 (0.037–0.090)	0.978 (0.960–0.993)	0.957 (0.923–0.987)
xload N (0, 0.005) res corr (d = 400)	0.283	0.038	−3.2	73.2	144	54	5531	0.071 (0.045–0.092)	0.973 (0.955–0.989)	0.951 (0.918–0.980)

Abbreviations: PIH, Partners in Health scale; PP p, posterior predictive *p*-value; # Par., number of free parameters; pD, estimated number of parameters; DIC, discrepancy information criterion; CFA, confirmatory factor analysis; xload, cross-loading; res corr, residual correlation; d, degrees of freedom.

**Table 3:** Bayesian models for CFA and using informative priors with cross-loadings and residual covariances.

		BSEM-CFA Four-Factor Model					BSEM with Cross-Loadings *N* (0, 0.005) and Residual Covariances IW(200*D,200)		
Item	K	P	M	C		K	P	M	C
					Loadings				
1	0.916 *	0	0	0		0.831 *	−0.019	0.037	0.013
2	0.918 *	0	0	0		0.869 *	0.024	−0.020	−0.011
3	0	0.915 *	0	0		−0.040	0.538 *	0.065	−0.060
4	0	0.517 *	0	0		0.057	0.772 *	−0.036	0.012
5	0	0.551 *	0	0		0.022	0.854 *	−0.072	0.012
6	0	0.836 *	0	0		−0.063	0.604 *	0.082	0.017
7	0	0	0.860 *	0		0.024	0.008	0.906 *	−0.023
8	0	0	0.885 *	0		−0.003	0.009	0.850 *	0.045
9	0	0	0	0.761 *		0.014	0.024	0.067	0.622 *
10	0	0	0	0.950 *		−0.001	−0.026	−0.070	0.971 *
11	0	0	0	0.964 *		−0.003	0.010	−0.019	0.915 *
12	0	0	0	0.523 *		−0.020	0.001	0.101	0.414 *
					Factor correlations				
K	-					-			
P	0.275 *	-				0.641 *	-		
M	0.350 *	0.842 *	-			0.409 *	0.662 *	-	
C	0.540 *	0.358 *	0.609 *	-		0.576 *	0.641 *	0.597 *	-

Abbreviations: BSEM, Bayesian structural equation model; CFA, confirmatory factor analysis; K, knowledge; P, partnership in treatment; M, recognition and management of symptoms; C, coping. Standardised values are presented. Values in bold indicate hypothesized major loadings. * *p* < 0.001.

## Data Availability

The original contributions presented in this study are included in the article. Further inquiries can be directed to the corresponding author.
